# Case of Uterine Polypus

**Published:** 1859-04

**Authors:** James Cody

**Affiliations:** Watertown, Wis.


### ARTICLE III.

### CASE OF UTERINE POLYPUS.

BY DR. JAMES CODY, OF WATERTOWN, WIS.

Widow C., aged 40 years, mother of four children, the youngest of which is now eight eight years old. She is of bilious nervous temperament; has always enjoyed uninterrupted good health until three years since, when the present malady made its appearance. I was called in haste to see her on the 28th of February last. On reaching the address, I learned the following history of the case: Three years since, and without any apparent cause, first experienced excessive menstruation; a year subsequent, it amounted to menorrhagia, or rather at that time it was pronounced menorrhagia; medical aid was sought at this time, and treatment instituted, and carried out, but no relief followed; the uterine hemorrhage was constant, and during the menstrual period, at various times, truly frightful and alarming, complete syncope coming on at short intervals. Present condition: pulse hardly perceptible at the wrist, and so quick as not to be counted; fainting and vomiting occurring very frequent; hands and feet cold; her countenance and general surface are ex-sanguine. Complains of all the cerebral symptoms consequent on hemorrhage. The legs and ankles are œdematous, and have been so for some time past. She is now flowing profusely. Digital compression of the abdominal aorta was resorted to for a few minutes, which checked the hemorrhage, and partial reaction took place. Arot. spirits ammonia, brandy; cold water dashed on the face; mustard poultice applied between the shoulders; heat applied to extremities. In a short time complete reaction took place. A vaginal examination now revealed a large pyriform polypus descend through the os uteri into the vagina and resting on the perineum, nearly, or quite filling the vaginal canal. The existence of such a disease was not dreamed of by the patient, her medical attendant, or her friends. The examination started the flowing again. The vagina was now plugged (as the patient was very weak, and could not bear the loss of any more blood; in fact, I thought she would surely die, the system became so bloodless), the T. bandage to retain it in place. Acet. plumbi. et opii. given in small and frequent doses. As she rallied up, nourishment and mild tonics given.

On March 3d, the patient feeling quite comfortable, the polypus was ligated; the hemorrhage, which for two days had been but slight, now completely ceased for nine hours, when it broke out again to an alarming degree; the abdominal aorta was again compressed with the fingers for a short time; stimulants were given, also the tampon was used during the night; she had frequent fainting spells and vomiting, but the hemorrhage was arrested.

Friday, 4th, 6 A. M.—Pulse, 130, small—tenetum aurem. No pain. Expresses herself as feeling quite well; some thirst; has vomited none since 3 o'clock; no fainting; extremities warm; flows a very little. The tampon was removed at 2 o'clock on account of pain. 1 P.M.—Feels comfortable, no pain; expresses herself as feeling better than for a month previous. The bladder was evacuated by catheter. The ligature was tightened half an inch or more; produced but slight pain.

9 P.M.—Reports herself as feeling very comfortable. Pulse 120, of good character. No flowing.

Saturday, 5th, 9 A.M.—Reports herself as feeling comfortable. Pulse 120. Rested well during the night.

Saturday, 5th, 1 P.M.—Ligature tightened; caused but slight and indistinct pain. Injected the vagina with soap suds.

9 P.M.—Has spent a good day.

Sunday, 6th, 9 A.M.—Reports as feeling better and stronger than for four months previous. Rested well during the night. Appetite good. Pulse 112. No dejection for three days. Ordered mag. calc. and seidlitz powders.

2 P.M.—Tightened ligature. Complained of slight pain in back, and in tumor.

Monday, 7th, 9 A.M.—Rested well during the night. Is quite comfortable. Pulse 116. No dejection. Is to have enema. Appetite good.

1 P.M.—Tightened ligature. Injected vagina with soap suds.

Tuesday, 8th, 1 P.M.—Rested well last night. Feels well to-day. Cathartic operated three times. Appetite good. Pulse 116. Tightened ligature. Injected vagina with soap suds.

Wednesday, 9th, 5 A.M.—Has suffered very much for two hours, in consequence of retention of urine. Pulse 120. Tenderness of abdomen. Urine drawn off. Abdomen fomented. Anodyne administered.

Wednesday, 9th, 2 P.M.—Feels better. No tenderness of abdomen. Tightened ligature. Complained of pain in back, also great soreness of vagina. Injected vagina.

9 P.M.—Retention of urine, causing considerable irritation of the system, and pain in the abdomen. Pulse 130. Used catheter; ordered fomentation to abdomen; spirits nit. dulc. et tr. opii., 60; tr. veratrum viride.

Thursday, 10th, 9 A.M.—Rested well during night. Pulse 120. Retention of urine. Used the catheter. On tightening the ligature and making slight traction, the canula came away.

The polypus was brought away pretty readily, although, from its size, some force and manipulation was necessary for its removal.

Friday, 11th, 9 A.M.—Reports herself as well.

Saturday, 12th, 9 A.M.—Is doing well.

Sunday, 13th, 9 A.M.—Complains this morning of headache and sickness at the stomach. Vomited some green matters. Ordered mag. calc. et rhei.

Monday, 14th.—Feels better this morning. Medicine operated well. Feels able to be up, and wishes to do so; consequently I permitted her to get up.

Tuesday, 15th, 9 A.M.—Is doing well. Was up yesterday.

Wednesday, 16th, 9 A.M.—Continues improving; up all day, yesterday. Appetite good. No vaginal secretion whatever. No bearing down. No pain in back. Head feels natural. No symptom whatever of uterine derangement. Says she feels as well as at sixteen years of age. The œdema of the legs and ankles is disappearing fast. The lips and cheeks are assuming their natural color.

April 2d.—To-day, I have visited this lady, and the change which has taken place in her system in the course of a fortnight is truly wonderful; comparatively from the verge of the grave to apparent good health. There can be no doubt but much of this lady's suffering and misery might have been ameliorated, or cured by an early examination, and consequently a true diagnosis of the malady; but such was not the case. Three years rolled round, and all that long period, in which the lady lost pounds and pounds of blood, the true condition of things was never dreamed of. The case was supposed to be a case of prolapsus uteri, attended with menorrhagia, and treated for such.

